# Perceived discrimination, mental health help-seeking attitudes, and suicide ideation, planning, and attempts among black young adults

**DOI:** 10.1186/s12889-024-19519-1

**Published:** 2024-07-29

**Authors:** Donte T. Boyd, Camille R. Quinn, Myles I. Durkee, Ed-Dee G. Williams, Andrea Constant, Durrell Washington, Sheretta T. Butler-Barnes, Aldenise P. Ewing

**Affiliations:** 1 College of Social Work, The Ohio State University, 1047 College RD, #325K, Columbus, OH 43215 USA; 2 Center for Equitable Family & Community Well-Being, School of Social Work, University of Michigan, Ann Arbor, MI USA; 3 Department of Psychology, University of Michigan, Ann Arbor, MI USA; 4 School of Social Work, Boston College, Chestnut Hill, MA USA; 5 Department of Sociology, The Ohio State University, Columbus, OH USA; 6 Crown School of Social Work, University of Chicago, Chicago, IL USA; 7 George Warren Brown School of Social Work, Washington University, St. Louis, MO USA; 8 College of Public Health, The Ohio State University, Columbus, OH USA

**Keywords:** Black young adults, Suicidal behaviors, Mental health help-seeking attitudes, Discrimination

## Abstract

**Background:**

Developing an understanding of the negative impact of discrimination is critical when examining the suicidality of Black young adults in the US. Suicide rates among Black young adults have increased at alarming rates. One of the reasons for this increase is the disparities related to access to mental health services, which has long-term health consequences. This study addresses a significant gap in the literature by examining associations between experiences of everyday discrimination, attitudes towards mental health help-seeking attitudes, on the outcomes suicide ideation, planning to die by suicide, and suicide attempts.

**Methods:**

The data came from a national study of the experiences of Black young adults regarding mental, physical, and sexual health. Participants were recruited from across the Midwestern region of the United States through Qualtrics Panels, an online survey delivery service used to recruit study participants. The total sample for this study was *N* = 362, and the average age of the sample was 21 (SD: 1.96). We used a logistic regression analysis to examine the role of everyday discrimination, mental health support-seeking attitudes, and covariates on the outcomes: suicide ideation, planning to die by committing suicide, and suicide attempts.

**Results:**

Black young adults with positive mental health help-seeking attitudes were 34% less likely to attempt suicide (*OR* = 0.66; 95% CI: 0.46, 0.96) and 35% less likely to experience suicide ideation (*OR* = 0.65; 95% CI: 0.47, 0.89). However, those young adults who experienced discrimination daily were more likely to report having attempted suicide (*OR* = 1.70; 95% CI: 1.34, 2.15).

**Conclusions:**

Our findings offer valuable insights into the complex interplay between experiences of discrimination, attitudes toward seeking mental health support, and suicidal behaviors. However, our research also underscores how experiences of discrimination can significantly exacerbate feelings of isolation, hopelessness, and inadequacy, further contributing to suicidal behaviors in this population. By promoting positive mental health help-seeking behaviors, actively addressing discrimination, and applying an intersectional approach to suicide prevention efforts, we can take significant strides towards building a more supportive and inclusive society. This approach aims to empower individuals to seek help, reduce the risk of suicidal behaviors, and create a more welcoming environment for all members of our community.

## Background

Suicide among youths aged 15 to 24 is a major concern for public health, and it is the third leading cause of death among this population in the US [1–3]. According to the National Survey on Drug Use and Health, from 2008 to 2019, suicide attempts significantly increased in number among 18 to 25-year-old young adults in the US [3]. Suicide rates among Black young adults aged 18 to 24 increased by 37% from 2018 to 2021 [4]. For other racial and ethnic groups, the Centers for Disease Control and Prevention (CDC) has reported a 16.7% increase for American Indian/Alaska Natives, a 10.6% increase for Asians, an 8.2% increase for Hispanics, and a 3.9% decrease for Whites [4]. Additionally, nonfatal suicidal behaviors such as thinking about suicide, planning to die by suicide, or attempting suicide have risen in frequency by 46% for 18 to 19-year-olds, 68% for those aged 20 to 21, 55% for 22 to 23-year-olds, and 29% for 24 to 25-year-olds [5]. These statistics are alarming, and unfortunately, there is a significant lack of representation of racial and ethnic minority individuals in suicide research [6, 7]. Perceived discrimination has been broadly linked to suicide ideation among Black adults regardless of age, gender, or socio-economic status. This study explores the complex relationship between mental health help-seeking attitudes and suicidal ideation, planning, and attempts among Black young adults. The goal is to identify the paths for offering professional help to young adults. Literature indicates an inordinate amount of underlying stigma, insufficient resources, as well as scant availability of resources that incorporate culture and religiosity. Our findings offer valuable insights into the complex interplay between experiences of discrimination, attitudes toward seeking mental health support, and suicidal behaviors.

### Mental health help-seeking attitudes

Research has shown that a complex relationship links suicide with attitudes toward seeking mental health support [8]. Many young people at risk of suicide, including those who die by suicide, receive no help from mental health services [8]. Black young adults face barriers to accessing mental health services (e.g., longer delays) compared to their White counterparts [8–10], with systemic racism playing a role through factors like income inequality and residential segregation. Other research exploring the MHSAs of Black young adults reveals an underutilization of available mental health services among those who experience pervasive mental health challenges [11–13]. Studies also report that while Black young adults underutilize mental health services, they often attempt to address their mental health needs independently and significantly rely on close family members and peers for mental and emotional support [14, 15], suggesting that Black young adults tend to adopt the self-reliance approach. However, recent data shows an alarming rise in suicide rates in this demography [3], and prior studies have found that seeking mental health services may coincide with the onset of suicidal behavior [8]. This highlights the importance of addressing barriers to mental health care access and promoting culturally relevant support for Black young adults.

The significant increase in suicide rates among Black youth underscores the critical importance of further examining their MHSAs. A more nuanced understanding of these attitudes is necessary, particularly regarding the underutilization of available mental health services. While help regarding mental health includes the utilization of both formal and informal mental health support, studies in this context have primarily focused on the use of formal mental health services while often ignoring the use of informal mental health supports, such as family members, peers, schoolteachers, religious leaders, and community members [16–18].

### Discrimination and suicidal behaviors

Discrimination is defined as negative actions and behaviors targeted at individuals or groups based on their marginalized social status [19, 20]. Previous studies have shown that Black Americans experience higher instances of subtle discrimination compared to Asian, Latinx, or White individuals [7], and discrimination is a chronic stressor for Black young adults [21]. In addition, research also indicates that experiences of discrimination are associated with numerous adverse mental health outcomes [18, 22–26]. These unfavorable outcomes include poorer mental health, psychological distress, trauma, violence, and poor self-esteem [18, 27]. Moreover, perceived discrimination has been broadly linked to suicide ideation among Black adults [28]. The results of a study on a nationally representative sample of Black adolescents indicated that racial or ethnic discrimination is associated with suicide ideation [29]. This was also observed in another study on racially and ethnically diverse groups in colleges [30]. However, findings from studies on this topic have not been consistent regarding the link between discrimination and suicidality among young Black adults. For instance, one study reported no direct relationship between discrimination and suicidal behaviors, suggesting that the relationship between these two elements is much more nuanced and warrants further investigation [31]. Developing an understanding of the negative impact of discrimination is critical when examining the suicidality of Black young adults in the US. Some studies have also noted that the collective history of different forms of discrimination against Black people should be recognized as a contributing factor when discussing suicidal behaviors and the need for and access to mental health care [31–34].

### Suicidal behaviors and black young adults

Overall, prior research has indicated that young adults exhibit higher levels of suicidal behaviors in comparison to other adult groups [35]. The existing literature regarding Black young adults in this context has focused on feelings of helplessness [1], self-blame [36], direct and indirect racism [36], and a sense of belonging linked to an increase in suicidal ideation [23]. Other forms of discrimination, associated with suicidal ideation, that Black young adults commonly face include exposure to racial microaggressions [37] and online racial discrimination [38, 39].

Other studies have observed that positive family and peer support is associated with a decreased risk of suicidal ideation and attempts [23, 40]. Some researchers have indicated that ethnic identity [41] and religiosity [3] protect Black young adults from suicidal behaviors. Unfortunately, most studies focusing on Black young adults and suicides have recruited participants from college samples [1, 36; 41, 42]. There is a dearth of studies that have examined MHSAs and everyday discrimination among a national sample of Black young adults aged between 18 and 24. While many studies have focused on the negative influence of discrimination on the mental health of Black youths [29–34], few have examined the relationship between discrimination, MHSAs, and suicidal behaviors.

### Mental health services

Suicide rates among Black young adults have increased at alarming rates. One of the reasons for this increase is disparities in access to mental health services, which has long-term health consequences. Black young adults may not seek mental health services due to the discrimination they face, which may influence their attitude toward the mental health system. This can create mistrust and potentially lead to avoidance of seeking help. However, when young people access mental health care services, it acts as a protective mechanism against suicidal behaviors [8]. This study fills a significant gap in the literature by examining the associations of everyday discrimination and MHSAs on the outcomes of suicide ideation, planning to die by suicide, and suicide attempts.

## Methods

### Procedures

In the current study, self-reported data were collected from a Midwestern study on young Black adult experiences with mental health services, perceived racism, and sexual health. Participants were recruited from across the United States through Qualtrics, an online survey delivery service used to recruit study participants, especially from difficult-to-reach populations [43]. The participants (ages 18 to 24) were selected through an email invitation from Qualtrics. They were eligible for the study if they identified as Black and were between 18 and 24 years of age. All eligible participants were required to provide consent before proceeding to take the survey. Once each individual gave their consent, they were permitted to proceed with completing the survey. The online survey took 20 to 30 min to complete. Ethical approval for the study was obtained from the Ohio State University Institutional Review Board (IRB # 2023E0165).

### Participants

Our sample comprised 362 self-identifying young adult Black men and women aged 18 to 24 (*M* = 21; *SD* = 1.96). The participants identified as Afro-American (81%), Afro-Caribbean/West Indian (10%), African (6%), and Afro-Latino (3%). 28% of the participants self-identified as male, 70% self-identified as female, and 2% identified as transwoman, transman, nonbinary, or other. 57% of the sample mentioned they were enrolled in school, and 34% were employed full-time. The majority of the sample reported having a high school diploma (41%), while 32% of the participants said they had a college degree, an associate degree, or a trade education degree. 34% of the participants had an annual household income of $19,000 or less. Most of the sample (75%) mentioned that they were living with their parents or a family member. The reported residence for about two-thirds of the sample included Ohio (28%), Illinois (20%), and Michigan (15%), while the other 37% lived across the Midwest. 64% of the participants had received health care in the previous six months, and 28% were covered by Medicaid.

### Measures

#### Dependent variables

*Suicide attempts* were measured using an item that asked the participants to indicate whether they had attempted to end their life within the last 12 months. *Suicide ideation* was measured using an item that asked the respondents to indicate whether they had considered ending their life over the previous 12 months. *Suicide planning* was measured using an item that asked the participants whether they had planned to end their life within the previous 12 months. For all questions, response categories were 1 = *yes* and zero = *no* [44].

#### Independent variables

*Everyday discrimination* was measured using the Everyday Discrimination Scale, which assesses daily experiences with discrimination [45–47]. The scale employs 10 items to assess the frequency of regularly occurring discrimination encounters related to being treated with less courtesy and respect; being provided poor restaurant service; being perceived as unsmart, dishonest, or not as good as others; and being intimidated, insulted, harassed, or followed in stores. These experiences were measured globally across all social identities (e.g., race, gender, and sexuality), and the response values for each item were zero = *never*, 1 = *less than once a year*, 2 = *a few times a year*, 3 = *a few times a month*, 4 = *at least once a week*, and 5 = *almost every day*. The Cronbach’s alpha for this scale was 0.88.

The Mental Health Help–Seeking Attitude Scale measures attitudes about seeking help from a mental health professional based on nine items and utilizing a 7-point Likert-type scale [48]. A total score was calculated by reverse-coding items 2, 5, 6, 8, and 9. Higher scores indicated more favorable attitudes toward seeking help from mental health professionals. The Cronbach’s alpha value for this scale was 0.90.

Several contextual variables were collected for the study as well. The participants indicated their age, income, and sex assigned at birth. Age and income were treated as continuous variables in the model, while sex assigned at birth was coded as zero for males and 1 for females.

### Statistical analysis

First, we examined the data for missingness among the main analytic variables and covariates. Table 1 presents the sample characteristics of categorical variables. Table 2 presents sample characteristics of continuous variables. Next, we conducted a bivariate logistic regression analysis (Table 3) between independent variables and covariates on study outcomes. Lastly, we used a multivariate logistic regression analysis (Table 4) to examine independent variables (everyday discrimination, MHSAs); covariates (sex at birth, age, income); and dependent variables (suicide attempts, suicide ideation, planning to die by suicide).

**Table 1:** Demographic variables (*N* = 362)

Demographic variable	*n*	Percent
**Gender**		
Male	100	28
Female	254	70
Transgender female	2	0.01
Transgender male	3	0.01
Nonbinary	14	0.04
Other	2	0.01
**Enrolled in school**		
Yes	202	57
No	152	43
**Education**		
College, postgraduate, or professional	30	8
Currently in school	32	9
High school diploma	144	41
Less than high school	10	3
Some college, AA degree, or trade school	114	32
Some high school	30	8
**Employment status**		
Homemaker	15	4
Employed for wages full time	105	30
Employed for wages part time	119	34
Not employed	75	21
Unable to work (disabled)	10	3
Self-employed	30	8
**Income**		
$0 to 19,000	115	34
$20,000 to $39,000	16	5
$40,000 to $74,999	2	1
$75,000 to $99,9999	7	2
$100,000 to $124,999	117	35
$125,000 to $149,000	2	1
$150,000 or more	6	2
**Health coverage in the past 6 month**		
Yes	230	64
No	132	36
**Health insurance type**		
Medicaid	100	28
Medicare	75	21
Plan through college or school	26	7
Private health plan from employer	43	12
Private health insurance plan purchased	21	6
Military, Champus, TriCare	4	1
Parents’ insurance plan	80	22
Other	13	4
**Suicidal behaviors**		
**Suicide Ideation**		
Yes	183	51
No	177	49
**Planned to die by suicide**		
Yes	130	36
No	230	64
**Suicide attempt**		
Yes	93	27
No	267	74
**Reasons for everyday discrimination**		
Race	163	45
Age	43	12
Gender	72	20
Sexual orientation	43	12
All other categories	–	–

**Table 2:** Continuous variables of study (*N* = 362)

Variable	M	SD	Range
Age	21	1.96	18–24
Mental health–seeking attitudes	4.37	0.76	1–7
Everyday discrimination	3.12	1.27	0–5

**Table 3:** Bivariate Logistic Regression (*N* = 362) on suicide ideation, planned to die by suicide, and suicide attempts

Variable	OR	SE	*P*-Value	95%CI
**Suicide ideation**				
Mental health-seeking attitudes	0.70**	0.10	0.012	0.52, 0.92
Everyday discrimination	1.47**	0.13	0.001	1.23, 1.76
Age	0.88**	0.05	0.04	0.79, 0.99
Income	0.94	0.04	0.24	0.85, 1.04
Sex assigned at birth (female reference)	0.61**	0.16	0.005	0.37, 1.01
**Planned to die by suicide**				
Mental health-seeking attitudes	0.69**	0.10	0.002	0.52, 0.93
Everyday discrimination	1.64***	0.16	0.001	1.36, 1.99
Age	0.99	0.06	0.94	0.88, 1.11
Income	0.90*	0.05	0.047	0.81, 0.99
Sex assigned at birth	0.99	0.28	0.98	0.56, 1.73
**Suicide attempts**				
Mental health-seeking attitudes	0.64**	0.11	0.008	0.46, 0.89
Everyday discrimination	1.60***	0.17	0.001	1.29, 1.97
Age	0.88	0.05	0.06	0.78, 1.00
Income	0.87***	0.05	0.03	0.78, 0.98
Sex assigned at birth (female reference)	0.89	0.26	0.69	0.49, 1.59

***Note***: *p < .05, **p < .01, ***p < .001; OR = Odds ratio; SE = Standard error; CI = confidence interval

**Table 4:** Multivariate Logistic Regression (*N* = 362)

Variables	OR	SE	*P*-Value	95%CI
**Suicide ideation**				
Mental health-seeking attitudes	0.65**	0.10	0.008	0.47, 0.89
Everyday discrimination	1.60***	0.16	0.001	1.31, 1.95
Age	0.82**	0.05	0.002	0.72, 0.93
Income	0.97	0.05	0.66	0.87, 1.08
Sex assigned at birth (female reference)	0.44**	0.12	0.005	0.25, 0.78
**Planned to die by suicide**				
Mental health-seeking attitudes	0.72*	0.12	0.043	0.52, 0.98
Everyday discrimination	1.61***	0.17	0.001	1.31, 1.98
Age	0.94	0.06	0.39	0.83, 1.07
Income	0.91	0.05	0.088	0.81, 1.01
Sex assigned at birth	0.99	0.28	0.98	0.56, 1.73
**Suicide attempts**				
Mental health-seeking attitudes	0.66*	0.12	0.031	0.46, 0.96
Everyday discrimination	1.70***	0.20	0.001	1.34, 2.15
Age	0.84*	0.06	0.025	0.73, 0.97
Income	0.89	0.05	0.075	0.79, 1.01
Sex assigned at birth (female reference)	0.63	0.21	0.18	0.32, 1.23

Note: *p < .05, **p < .01, ***p < .001; OR = Odds ratio; SE = Standard error; CI = confidence interval

## Results

In the previous 12 months, approximately half (51%) of the sample self-reported experiencing suicide ideation, with 36% stating that they had planned to commit suicide in the previous 12 months, and 27% reported attempting suicide (Table 1). Black young adults reported higher than average positive MHSAs (*M* = 3.27; *SD* = 0.76) and experiences of daily discrimination (*M* = 3.12; *SD* = 1.27) (Table 2).

### Bivariate regression analysis

Black young adults with positive MHSAs were 30% less likely to think about suicide (OR = 0.70; 95%CI: 0.52, 0.92; *p* = .008), 31% less likely to plan committing suicide (OR = 0.69; 95%CI: 0.52, 0.93; *p* = .002), and were 36% less likely to attempt suicide (OR = 0.64; 95%CI: 0.46, 0.89; *p* = .008) in comparison to young adults who had negative mental health support-seeking attitudes in the past 12 months. Young adults who had experienced discrimination daily were 47% more likely to think about suicide (OR = 1.47; 95%CI: 1.23, 1.76; *p* < .001), 64% more likely to plan to die by suicide (OR = 1.64; 95%CI: 1.36, 1.99; *p* < .001), and were 60% more likely to attempt suicide in comparison to Black young adults who did not experience daily discrimination (OR = 1.60; 95%CI: 1.29, 1.97; *p* < .001) in the past year. Lower-income young adults were 10% less likely to plan to die by suicide (OR = 0.90; 95%CI: 0.81, 0.99; *p* = .047) and 13% less likely to attempt suicide (*OR* = 0.88; 95%CI: 0.78, 0.98; *p* = .03) in comparison to higher earning young adults in the past year (Table 3).

### Multivariate logistic regression analysis

Black young adults with positive MHSAs were 35% less likely to experience suicide ideation (*OR* = 0.65; 95% CI: 0.47, 0.89; *p* = .008), 28% less likely to plan to die by suicide (*OR* = 0.72; 95% CI:0.52, 0.98; *p* = .043), and were 34% less likely to attempt suicide (*OR* = 0.66; 95% CI: 0.46, 0.96; *p* = .031) in comparison to young adults who had negative mental health help-seeking attitudes in the past year. Young adults who experienced discrimination daily were associated with an increase in experiencing suicide ideation (*OR* = 1.60; 95% CI: 1.31, 1.95; *p* < .001), planning to die by suicide (*OR* = 1.61; 95% CI: 1.31, 1.98; *p* < .001), and attempting suicide (*OR* = 1.70; 95% CI: 1.34, 2.15;*p* < .001) in comparison to Black young adults who did not experience daily discrimination in the past 12 months. Younger Black adults were 18% less likely to experience suicide ideation (*OR* = 0.82; 95% CI: 0.72, 0.93; *p* = .002), and 16% less likely to attempt suicide (*OR* = 0.84; 95% CI: 0.73, 0.97; *p* = .025) in comparison to older individuals in the past year. Black men were 56% more likely to experience suicide ideation than women (*OR* = 0.44; 95% CI: 0.25, 0.78; *p* = .005) in the past year (see Table 4).

## Discussion

The present study examined whether everyday discrimination and mental health help-seeking attitudes were associated with suicide ideation, planning to die by suicide, and suicide attempts among Black young adults aged 18 to 24. Our findings offer valuable insights into the complex interplay between experiences of discrimination, attitudes toward seeking mental health support, and suicidal behaviors. In our sample, 51% of Black young adults experienced suicide ideation, 36% had planned to die by suicide, and 27% reported attempting suicide. This study links discrimination to planning to die by suicide and suicide attempts. Further, this study underscores the importance of seeking help for mental health to reduce suicidality. The results also indicated that Black men were more likely than Black women to experience suicide ideation. Consequently, this further supports previous research, indicating that suicide rates among Black young adults have steadily increased over the past decade [49–52].

### Mental health help-seeking attitudes and suicidality

Our findings suggest that MHSAs are associated with decreased suicide ideation, planning to die by suicide, and attempts among Black young adults. This is critical because seeking help for mental health concerns is essential for overall well-being and quality of life [53, 54]. Previous research has indicated that providing mental health services to young people can increase their likelihood of engaging with and utilizing these services [8]. Research has also indicated that positive attitudes toward seeking mental health support, such as acceptance, openness, and willingness to seek help, are associated with a greater likelihood of seeking support when experiencing suicidal ideation [8]. This study also expands the literature by examining how MHSAs decreased planning to die by suicide and suicide attempts. Exploring the relationship between mental health help-seeking attitudes and suicide planning among Black young adults is crucial for understanding the unique challenges and factors influencing suicidal behaviors within this population.

Unfortunately, due to structural racism, i.e., the role of the structures (laws, policies, institutional practices, and entrenched norms) that are the systems’ scaffolding [55], stigma, interpersonal discrimination, and the like, Black young adults are at risk of committing suicide. Due to structural racism, they face barriers while accessing mental health services (e.g., longer delays) compared to their White counterparts [8–11], with systemic racism playing a role through factors like income inequality and residential segregation. Other scholars note that lower health/mental health service utilization is associated with a general mistrust of these providers; our study sample may likely hold similar views [56, 57]. Addressing these barriers requires a comprehensive approach that includes promoting mental health literacy, offering culturally responsive and accessible mental health services, and engaging with the community to foster positive attitudes toward seeking mental health support among Black young adults.

Having a positive attitude toward seeking help for mental health challenges and suicide is crucial for the overall well-being and quality of life of Black youth. Research indicates that a positive mindset about seeking support can lead to improved mental health outcomes and lower suicidality. However, research has revealed that it is critical to pay attention to social support across different ecological contexts where young adults live, learn, and socialize [58]. Within these different contexts, clinicians, families, and other supportive individuals should encourage open discussions about mental health and suicidality. The results of a study implied that the compounding effects of social support from family and schools lowered suicidality [58]. Compounded social support from individuals who share similar cultural backgrounds to Black young adults can provide a sense of understanding and validation of the unique challenges faced by this population. This can potentially help reduce feelings of isolation and stigma associated with seeking help for suicidal thoughts and behaviors. Incorporating appropriate services addressing culture and religiosity is an effective strategy to eliminate the stigma of help-seeking for young Black adolescents and young adults. Empowering Black young adults to take a proactive approach can result in improved mental health outcomes, strengthened coping skills, and greater self-efficacy in managing stress and emotional challenges [59, 60]. Encouraging Black young adults to actively engage in their mental health and to seek support when needed can potentially empower them to develop resilience and effective strategies for navigating difficult situations, potentially reducing the risk of suicidal behaviors.

Clinicians, social workers, and other practitioners play a crucial role in providing guidance, resources, and support to help individuals build the skills and coping mechanisms necessary to overcome challenges and prioritize their mental well-being. Compounded social support can offer coping strategies that are culturally relevant and effective in managing their mental health challenges. This can include strategies, such as communal coping, spiritual resilience, and drawing on cultural strengths and traditions. Through early intervention and support, professionals can empower individuals to develop effective coping strategies, build resilience, and prioritize their mental well-being.

### Everyday discrimination and suicidality

Our results highlight the significant impact of everyday discrimination, which was associated with an increased likelihood of suicide ideation, planning, and attempts. Similar to the findings of previous research, participants reporting higher discrimination levels in our study were more likely to report elevated suicide risks [24]. These findings align with prior research, indicating that encountering discrimination is positively related to the severity of suicide ideation within the past two weeks for Black young adults [44]. This study also makes a significant contribution by linking discrimination to an increase in planning to die by suicide among Black young adults. It is possible that facing discrimination daily can contribute to feelings of hopelessness, despair, and worthlessness, placing Black young adults at a higher risk for mental health issues, such as depression and anxiety [40]. In turn, these mental health challenges can increase the likelihood of planning to die by suicide.

However, the factors and processes involved in choosing a particular method of suicide can be complex and not well-understood, especially when it comes to Black young adults. While research has shown the impact of discrimination on mental health outcomes and suicide risk, there is a need for further study to better understand the factors that influence the planning to commit suicide. Our results also indicated that discrimination increased the likelihood of Black young adults attempting suicide. This is consistent with the current research results that different forms of daily discrimination increase suicide attempts [33]. For Black young adults, the cumulative effects of systemic racism, interpersonal discrimination, and marginalization can contribute to increased feelings of emptiness, which are the risk factors for suicidal attempts.

Discrimination against Black young adults concerning suicide prevention and mental health support is a pressing concern that needs addressing. These people face unique challenges and systemic barriers when seeking mental health services, due to different forms of discrimination [37–39] contributing to disparities in access when compared to their White counterparts. It is essential to recognize and work to dismantle discriminatory practices and biases hindering Black young people from receiving the assistance needed to address mental health concerns and to prevent suicide. Efforts to promote culturally competent and inclusive mental health services are crucial to ensuring that all individuals, regardless of race or ethnicity, receive the support and care they deserve. This highlights the cascading effects of discrimination on suicidal behaviors, emphasizing the need for targeted interventions at multiple levels. Thus, it is imperative to identify factors linked to the progression from suicide ideation to suicide attempts, as such attempts serve as predictors of eventual suicide mortality [61, 62].

### Gender and suicidality

Within our sample, Black men were more likely to report an increase in ideation of suicide than Black women. This is consistent with prior literature showing increased suicidal behaviors among Black men over the past two decades [63–65]. In addition, epidemiological data indicate that Black men in the US across all age groups die by suicide at rates 4 to 6 times higher than Black women [64]. Nevertheless, the current discourse on suicide prevention predominantly centers around non-Latinx White culture [66, 67]. This focus can render suicidality in Black youth “invisible” [22]. This exclusionary framing underscores the need to broaden the conversation and incorporate diverse cultural perspectives, particularly those of Black men, in suicide prevention efforts. Acknowledging and addressing these disparities can help us work toward more inclusive and effective strategies to support the mental health and well-being of Black men.

### Limitations

While our study contributes to a deeper understanding of the mechanisms linking discrimination and attitudes toward MHSAs to suicidal outcomes, several limitations should be acknowledged. We did not measure the timing of discrimination experiences; thus, we cannot determine whether they occurred before or after reported instances of suicidal ideation, planning, or attempts. Additionally, the cross-sectional nature of the data limits our ability to draw causal inferences about the observed relationships. The reliance on self-reported measures may introduce biases and limitations associated with subjective reporting. Although we did not use a single measure for suicide (we included measures of suicide ideation, planning, and attempts), we used single items for each of these variables, limiting the rigor of our analyses.

Despite these limitations, our study contributes to the literature by focusing on an underserved, understudied, and vulnerable population. Future research should include longitudinal studies to explore temporal dynamics and causal pathways involved in these associations over time. Additionally, studies should incorporate multimethod approaches and mental health outcomes for a more comprehensive understanding [68].

### Implications for research, policy, and practice

The findings of our study have significant implications for clinical practice, public health initiatives, and policy interventions aimed at promoting mental health and preventing suicide. Experiences of discrimination could vary by gender and across races, indicating additional within-group diversity that may differentially impact suicide-related risk [69]. Addressing the root causes of discrimination and creating supportive environments fostering inclusivity and acceptance are crucial steps toward reducing the burden of mental health disparities and enhancing access to care for marginalized and vulnerable populations.

Furthermore, our study underscores the importance of comprehensive suicide prevention efforts extending beyond traditional risk assessment and intervention strategies. One way to achieve this is by capturing the collective effects of discrimination on suicide risk [68]. This approach would probably enhance the ability to recognize the role of social determinants, such as discrimination, in shaping individuals’ MHSAs and mental health experiences. This is essential for developing holistic approaches to suicide prevention that address underlying social and structural factors.

Research consistently shows that experiences of discrimination, especially racial discrimination, can have detrimental effects on mental health outcomes [70, 71]. For Black youth already facing societal and systemic challenges, everyday discrimination can exacerbate mental health issues [72, 73]. Discrimination can result in elevated stress levels, reduced self-esteem, and a detrimental impact on overall mental well-being [74]. These adverse effects may manifest in various ways, including heightened symptoms of depression, anxiety, and in extreme cases, suicide ideation, planning, and attempts.

To address the alarming rates of suicide, it is crucial to implement interventions and policies promoting mental health awareness, reducing stigma, and providing culturally competent care. Creating safe and inclusive spaces where Black young adults feel supported and understood is essential. Offering accessible, affordable, and tailored mental health resources and services can encourage help-seeking attitudes and reduce the risk of suicide ideation, planning, and attempts. Addressing and reducing everyday discrimination also is a crucial step in improving the mental health outcomes of Black young adults. This can be achieved by implementing antidiscrimination policies, promoting inclusivity and diversity, and educating individuals and communities about the impact of discrimination on mental health and well-being. The implications of everyday discrimination on MHSAs, suicide ideation, planning, and attempts among Black young adults aged 18 to 24 are significant. Addressing discrimination, promoting mental health awareness, and providing culturally competent care are the key steps toward mitigating the negative impact on mental health outcomes for this population.

## Conclusion

Our research emphasizes the significance of MHSAs and the influence of discrimination on suicidal behaviors. Research indicates that experiences of discrimination can pose obstacles to accessing mental health services for individuals from racial and ethnic minority groups [75]. The fear of judgment, stigma, and a lack of culturally competent care can dissuade Black young adults from seeking help, exacerbating their mental health struggles, and potentially leading to higher rates of suicidal behaviors. Addressing the root causes of mental health inequalities and advocating for inclusive, culturally sensitive care can establish fairer and more nurturing environments that promote psychological health across all communities. As we consider future research, examining the strengths of Black young adults, as opposed to deficits, can provide valuable insights.

## Data Availability

The data for this study will be made available upon written request to the corresponding author detailing the specific parts of the data to be shared and the intended purpose.
